# Surface plasmon resonance based sensor for the detection of glycopeptide antibiotics in milk using rationally designed nanoMIPs

**DOI:** 10.1038/s41598-018-29585-2

**Published:** 2018-07-25

**Authors:** Zeynep Altintas

**Affiliations:** Institute of Chemistry, Technical University of Berlin, 10623 Berlin, Germany

## Abstract

Glycopeptide antibiotics are known as the last resort for the treatment of serious infections caused by Gram-positive bacteria. The use of milk products contaminated with these antibiotic residues leads to allergic reactions and sensitivity in human. Also, long-term consumption of milk products containing low levels of these antibiotics may cause the relevant bacteria to build up resistance to these last resort antibiotics. Sensitive, rapid and effective quantification and monitoring systems play a key role for their determination in milk products. Hence, molecularly imprinted nanostructures were rationally designed in this work to produce high affinity synthetic receptors to be coupled with a surface plasmon resonance sensor for the analysis of glycopeptide antibiotics in milk samples. The nanoMIP-SPR sensor enabled vancomycin quantification with the LODs of 4.1 ng mL^−1^ and 17.7 ng mL^−1^ using direct and competitive assays, respectively. The recoveries rates for two sensor methods ranged in 85–110% with RSDs below 7%. The affinity between the nanoMIP receptors and the target molecule (dissociation constant: 1.8 × 10^−9^ M) is mostly superior to natural receptors and other synthetic receptors. Unlike other methods commonly employed for the detection of milk contaminants this approach is extremely simple, fast and robust, and do not require pre-sample treatment.

## Introduction

Glycopeptide antibiotics are known as the last resort for the treatment of life-threatening diseases caused by Gram-positive bacteria, such as *Clostridium difficile*, *Enterococcus* spp. and *Staphylococcus aureus*. Various groups of soil actinomycetes are responsible from the production of these glycosylated non-ribosomal peptides. Glycopeptide antibiotics target Gram-positive bacteria by binding to the acyl-d-alanyl-d-alanine (d-Ala-d-Ala) terminus of the growing peptidoglycan on the outer cell membrane surface. Microorganisms gained resistance to these glycopeptides avoid such a fate by replacing the d-alanyl-d-serine (d-Ala-d-Ser) or d-Ala-d-Ala terminus with d-alanyl-d-lactate (d-Ala-d-Lac), thus notably lowering antibiotic affinity for the cellular target. The use of antibiotics on farm animals may cause allergic reactions and chemical poisoning leading to the transfer of these compounds into to food chain^1^. The overuse of antibiotics can result in the development of bacterial resistance causing insufficient or unsuccessful treatment of human or animal diseases^2,3^. The presence of these pharmaceuticals in milk also affects the fermentation process and this has resulted in intensive regulations on milk to protect public health^4^. Maximum residue levels (MRLs) of antibiotics allowed to be found in milk are in trace amounts that range in 10–200 µg kg^−1^ ^5^. There is a need to develop highly sensitive quantification methods that should also be rapid, easy-to-use and reliable. Several techniques exist for pharmaceuticals’ determination such as spectroscopy^6,7^, liquid chromatography-mass spectroscopy (LC-MS)^8,9^, high performance liquid chromatography (HPLC)^10,11^, capillary electrophoresis^12^ and sensors^5,9,13–15^. Sensor technologies offer clear advantageous when compared to the other methods such as easy handling, rapid analysis without pre-sampling, and high sensitivity of assay platforms that allow detecting trace amounts of antibiotics in milk. Effective detection principles using different sensor systems have been reported for pharmaceutical quantification and monitoring^9,13,15,16^. The integration of nano-molecularly imprinted polymers (nanoMIPs) as synthetic affinity receptors into sensor devices has made a significant contribution to the bio-detection field. NanoMIPs produced using a solid phase has been pioneered by our group^9,17,18^ and the solid phase synthesis approach demonstrates superior advantages over the other MIP synthesis methods^17–21^: Low affinity polymers can be discarded during the manufacturing process by applying cold wash (1), high affinity nanoMIPs are collected by performing elution steps at high temperature (2), the template free receptors are obtained with this method, because nanoMIPs are removed from the template molecule rather than the template being removed from the MIP (3), the stability and size of the nanoMIPs are high owing to the selective washing and elution steps (4), a significant amount of MIPs can be obtained per synthesis that is adequate for long term experiments (5)^17–21^. These superior features have resulted in the successful applications of nanoMIPs in various fields such as pharmaceutical monitoring^9,13^, virus quantification^18,21^, endotoxin detection^22,23^, development of enzyme linked immonosorbent assays (ELISA)^24,25^, manufacturing of bioselective membrane filters^11^, and *in vivo* recognition of biomarkers^26^.

The current work aims to extend the applications of nanoMIPs in food samples analysis to determine the presence of glycopeptide antibiotics in milk. NanoMIPs targeting vancomycin as a natural glycopeptide were therefore rationally designed by employing computational simulations and they were synthesized using the best polymer composition. The synthetic receptors were then used for the construction of an optical sensor for the sensitive detection of vancomycin in milk samples. To the best of our knowledge this is the first computationally designed nanoMIP for milk sample analysis using sensors. More importantly, this work provides the most sensitive and rapid analysis using the nanoMIP receptors and it introduces two different sensor assays to effective milk sample analysis for the first time: nanomaterial-conjugated direct assay and competitive assay.

## Results and Discussions

### Rational Design of NanoMIPs for Glycopeptide Antibiotic

The computational modelling software allows visualisation of binding interactions of the monomers with the vancomycin template (Supplementary information, Figs S1–S6). The modelling approach eliminates unsuitable monomer candidates and narrows down the selection of monomers that enormously reduce the real application time in the laboratory and the required cost for research^13,22^. Moreover, the use of monomers competing for the same binding site when two functional monomers are used in a recipe can be avoided by visualising the molecular interactions between the target molecule and each monomer^27^. The individual monomers were docked to vancomycin target to calculate the binding energies and the highest binding energy was recorded for itaconic acid as the best functional monomer with −50.71 kcal mol^−1^ (Table 1). The highest negative binding energy scores represent the high affinity between the template and functional monomers.

**Table 1:** Molecular modelling results depending on binding energy between the monomer and vancomycin target.

Monomer	Abbreviation and charge of monomer	Binding energy (kcal mol^**−**1^)
Itaconic acid	−IA	−50.71
Trifluoromethacrylic acid	−TFMAA	−44.33
Methacrylic acid	−MAA	−43.41
Urocanic acid	−UA	−43.36
Ethylene glycol methachrylate phosphate	EGMP	−40.60
Diethylaminoethyl methacrylate	+DEAEM	−40.00
Hydroxyethyl methacrylate	HEM	−39.63
Methylene-bis-acrylamide	MBAA	−39.50
Acrylamide	Acrylamide	−37.67
Urocanic acid	UA	−34.44
Urocanic ethyl ester	UAEE	−32.00
1-vinylimidazole	+VI	−30.90
Ethylene glycol dimethacrylate	EGDMA	−28.55
Trifluoromethacrylic acid	TFMAA	−25.61

### NanoMIP Synthesis and Characterisation

**Figure 1:**
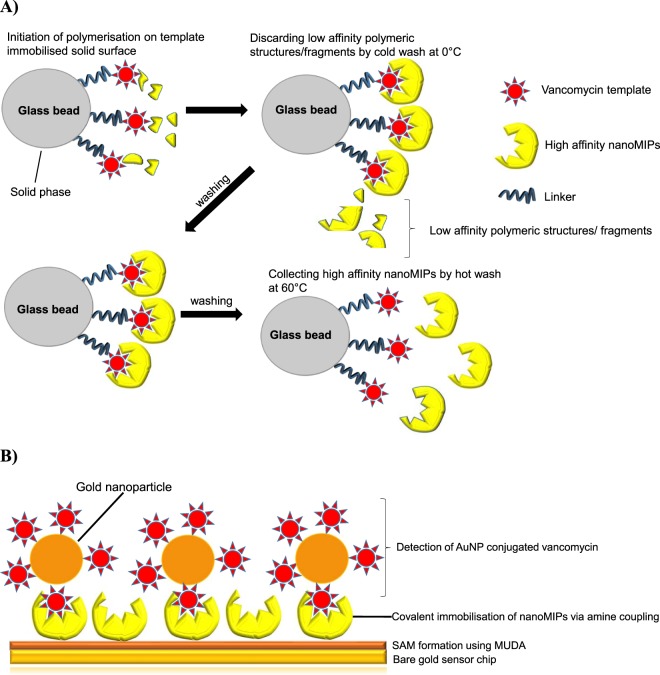
Schematic illustrations of the preparation of vancomycin nanoMIPs (**A**) and their use in sensor development as synthetic affinity receptors for vancomycin detection (**B**).

The high affinity nanoMIPs were manufactured using solid phase synthesis method where vancomycin template was initially immobilised on the solid phase (Fig. 1A). The polymer mixture was poured on the glass beads and then exposed to UV light to perform the polymerisation. Low affinity polymers were discarded by applying cold wash and the high affinity nanoMIP receptors were collected by carrying out hot wash in the last step.

**Figure 2:**
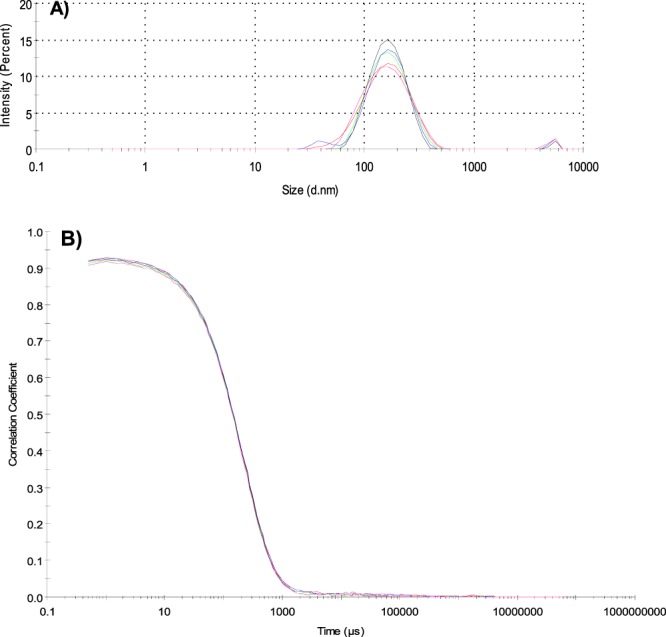
Size distribution analysis of nanoMIPs by intensity (**A**) and correlation analysis of data of four separate nanoMIP productions (**B**) using DLS method.

The size and quality determination of the synthesised vancomycin nanoMIPs were carried out using the dynamic light scattering (DLS) method. The nanoMIPs produced as four separate batches were measured and their average size was found to be 174 ± 2.16 nm with a polydispersity index of 0.2. The lower PDI value is an indication for high quality and uniform nanoMIP samples^9^. The size and PDI values of each separate synthesis are given in Table 2. The correlation of raw data, data fit and size distribution by intensity were also evaluated to assess the reproducibility and reliability of the DLS results (Fig. 2). The correlation between the data of four separate syntheses confirms the consistency and stability of the nanoMIP receptors.

**Table 2:** Size, yield and affinity determination of nanoMIPs.

NanoMIP	Diameter (nm)	Polydispersity (PDI)	Yield (mg)	Dissociation constant (K_d_)
Batch 1	174 ± 9	0.2	15.2	1.3 × 10^−9^ M
Batch 2	176 ± 11	0.2	14.5	2.4 × 10^−9^ M
Batch 3	171 ± 5	0.2	15.6	1.1 × 10^−9^ M
Batch 4	175 ± 7	0.2	14.7	2.7 × 10^−9^ M

Size of nanoMIPs and PDI values were determined using DLS. Affinity between nanoMIPs and vancomycin target is expressed as dissociation constant, which was calculated using Biacore 3000 analyzer.

To determine the yield per production the vancomycin nanoMIP samples were concentrated by evaporating excess solvent under heating while agitating the solution with nitrogen gas. The total yield for each batch of nanoMIP production was found to be around 15 mg that is a significant amount that allows conducting characterisation studies and sensor assays for a long time (Table 2).

### NanoMIP-SPR Sensor for Vancomycin Detection in Milk

**Figure 3:**
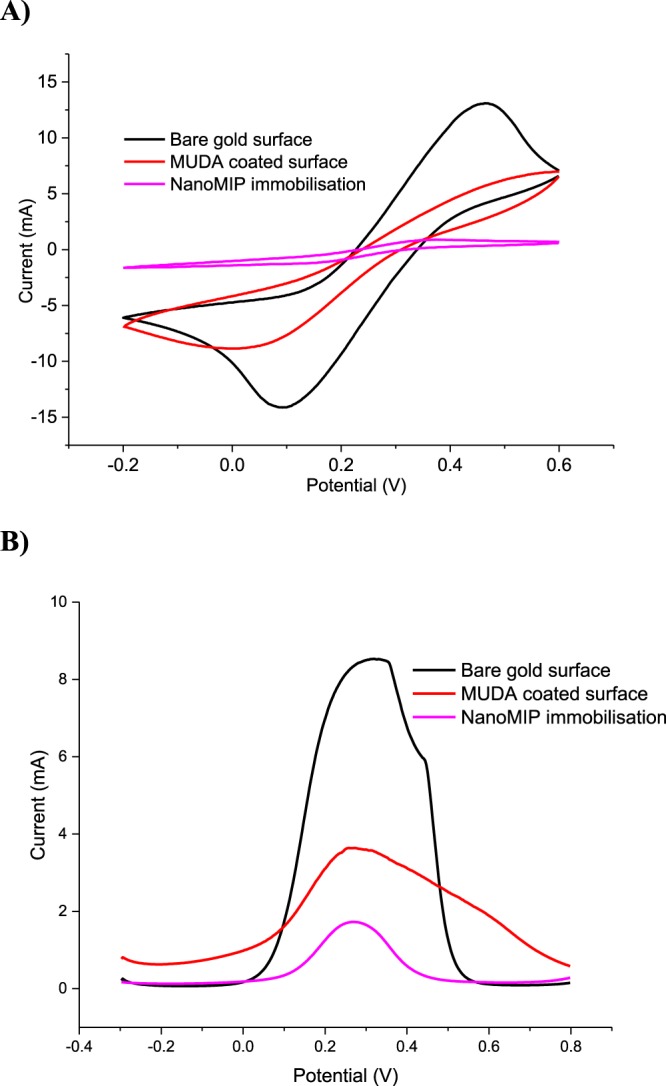
Sensor surface characterisation using voltammetry techniques. Cyclic (**A**) and square wave voltammograms (**B**) recorded in 0.1 M K_3_Fe(CN)_6_ and 1 M KCI for bare gold chip (black curve), MUDA coated sensor chip surface (red curve) and nanoMIP immobilised sensor surface (pink curve).

NanoMIP-SPR sensor was prepared by covalently immobilising the nanostructured polymeric receptors on the chip surface via amine coupling chemistry^18,28^. The surfaces of bare gold, thiol coated (11-mercaptoundecanoic acid: MUDA) and nanoMIP immobilised sensor chips were characterised using voltammetry techniques^29^. The ferrocynanide signal was largely suppressed after self-assembled monolayer formation using thiol solution indicating the successful coating of the chip with MUDA. The peak heights of cyclic voltammetry (CV) curves further decreased after nanoMIP immobilisation (Fig. 3A). The results of CV were also confirmed by square wave voltammetry (SWV) measurements that demonstrated SWV peaks of 8.5 mA, 3.5 mA and 1.5 mA for bare, MUDA coated and nanoMIP immobilised surfaces, respectively (Fig. 3B).

**Figure 4:**
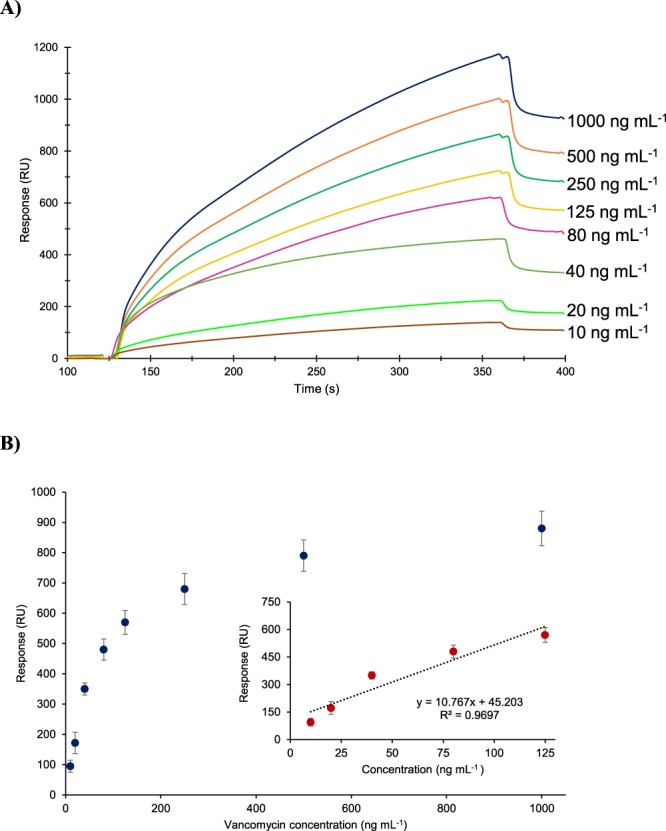
Concentration dependent real time sensorgram (**A**) and overall results of vancomycin detection assays in the range of 10–1000 ng mL^−1^ along with the linear regression analysis of the data revealed a R^2^ value of 0.96 (**B**).

The initial bio-detection studies were carried out using free vancomycin without gold nanoparticle (AuNP) conjugation. This assay resulted in a detection limit of 50 μg mL^−1^ that is not sufficient enough to quantify the trace amounts of glycopeptide antibiotics in milk samples. To increase the sensitivity of the bio-detection assays the vancomycin target was conjugated with AuNPs. The samples were prepared in skimmed milk in the concentration range of 5–1000 ng mL^−1^. The concentration dependent real time sensorgram of vancomycin assay is given in Fig. 4A and the overall results of vancomycin quantification is shown in Fig. 4B along with linear regression analysis, providing a R^2^ value of 0.96 that confirms the reproducibility of the assays. The LOD was verified based on the linear portion of the saturation graph by calculating three times standard deviation of the blank response (29.9 × 3 = 89.7 RU) and extrapolating the sensor signal in the linear calibration curve to convert the value to concentration. The limit of detection was determined to be 4.1 ng mL^−1^ with a linear range from 10 ng mL^−1^ to 125 ng mL^−1^ as shown in Fig. 4B (inset). The sensitivity of nanoMIP-SPR sensor is sufficiently high for antibiotic quantification as MRL for antibiotics in milk ranges from 10 to 200 µg kg^−1^.

Furthermore, the kinetic data analysis was performed using Biacore 3000 analyzer to determine the affinity between the synthesised nanoMIPs and the vancomycin target. The calculation of dissociation constant (K_d_) for each production of nanoMIPs was demonstrated in Table 2. The average dissociation constant was found to be around 1.8 × 10^−9^ M that indicates a high affinity between the nanoMIP receptors and vancomycin target. The affinity of vancomycin nanoMIPs is in good agreement with the previously reported drug nanoMIPs (1.48 × 10^−9^ M for diclofenac; 1.35 × 10^−10^ M for metoprolol) that were used as synthetic receptors for drug monitoring in drinking water^9,13^. These sensors allowed quantifying diclofenac and metapolol down to 1.24 ng mL^−1^ and 1.9 ng mL^−1^ using nanoparticle-conjugated assay approach, respectively. However, the matrix effect of drinking water is far less than milk samples and the production of MIPs for these drugs is easier due to their smaller size and less complex structures when compared to vancomycin.

The detection of other antibiotics in five times diluted milk samples was reported using a portable SPR sensor that relied on haptenized proteins as bioreceptors^5^. The haptenized proteins were covalently attached to the sensing surface by means of a previously formed self-assembled monolayer. The sensor could quantify enrofloxacin, sulfapyridine and chloramphenicol in the concentration ranges of 0.05–719 μg L^−1^, 0.03–499 μg L^−1^, and 0.04–647 μg L^−1^ with LOD of 1.7 µg mL^−1^, 2.1 μg L^−1^, and 1.1 μg L^−1^, respectively. The sensitivity of this sensor is significantly lower than that of nanoMIP-SPR sensor in the current study as the authors used direct assay strategy without nanomaterials that did not allow detecting maximum residue levels of some antibiotics (i.e. chloramphenicol)^5^. Other works carried out using Biacore instruments resulted in similar detection limits after the removal of matrix fat^30^. The sensitivity of sensor systems can be further increased up to 0.1 μg kg^−1^ when sample treatment or clean-up steps are applied^31^. Nevertheless, this approach still lacks of desired sensitivity and the need for nanoparticle-modified assay principle is clear.

**Figure 5:**
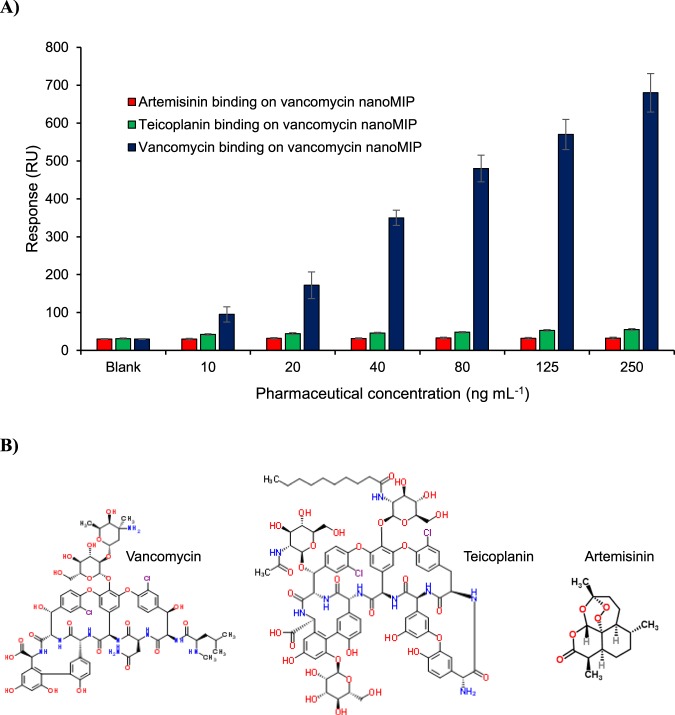
Cross-reactivity analyses in the concentration range of 10–250 ng mL^−1^ using the target (vancomycin) and control drugs (artemisinin and teicoplanin). Blank represents the background signal of skimmed milk without any drug molecule (**A**). Chemical structures of three drugs used in this work (**B**).

The cross-reactivity of non-specific drug molecules (teicoplanin and artemisinin) to vancomycin nanoMIPs was also accessed along with the background signal of the blank sample (skimmed milk without analytes) (Fig. 5A). The target and control drug samples prepared under the same conditions were injected to the vancomycin nanoMIP immobilised sensor surfaces at the concentration range of 10–250 ng mL^−1^. Teicoplanin is another glycopeptide antibiotic that shows structural similarity to vancomycin (Fig. 5B). However, its interaction with the nanoMIP surface produced maximum 55 ± 2.4 RU response (corresponding result of 250 ng mL^−1^ teicoplanin sample injection), suggesting the specificity of the receptor cavities for vancomycin target in terms of shape, size and chemical functionality. It is worth to mentioning that around 30 ± 2.4 RU sensor signal was recorded as a result of blank sample injection, meaning that only 25 RU signal change was produced upon the binding of teicoplanin on the nanoMIP immobilised surface for the highest concentration of the drug. Artemisinin is a small antimalarial drug that revealed a maximum signal of 32.5 ± 2.5 RU (corresponding to 250 ng mL^−1^ of artemisinin) upon interacting with the nanoMIP surface. This is most likely caused by much smaller size and considerably different structure of artemisinin when compared to vancomycin (Fig. 5B). The background signal of blank sample was also evaluated and it was found to be 30.3 ± 2.4 RU that was around three times lower than the signal of 10 ng mL^−1^ target binding.

**Figure 6:**
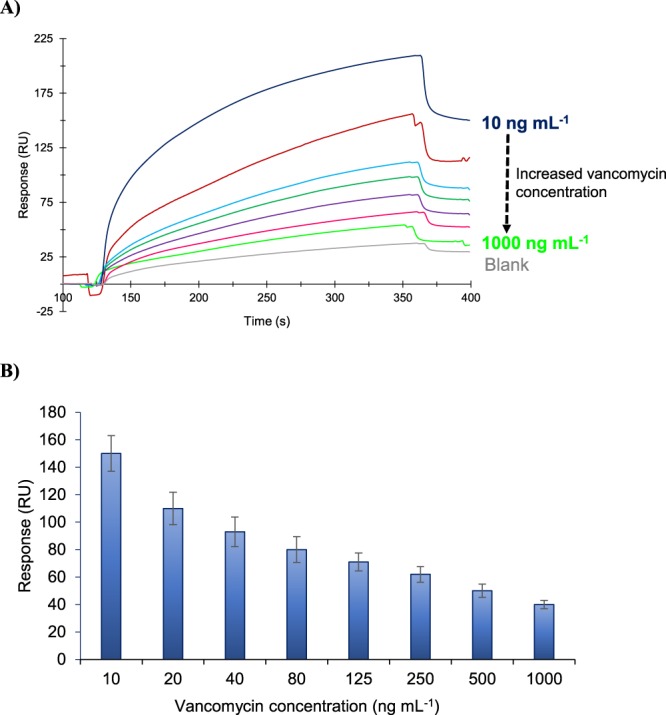
Concentration dependent real time sensorgram (**A**) and overall results of competitive assay (**B**) in the concentration range of 10–1000 ng mL^−1^ (n = 3).

A competitive bioassay was also performed in this work by mixing AuNP conjugated vancomycin and free vancomycin samples in skimmed milk to investigate the competition between vancomycin and AuNP-vancomycin. Eight different concentrations of vancomycin (10, 20, 40, 80, 160, 320, 500 and 1000 ng mL^−1^) in separate Eppendorf tubes were used and each sample was injected onto vancomycin-nanoMIP immobilised sensor surfaces for 4 min. A gradual decrease in sensor signal was observed with the increased concentration of free vancomycin molecules, indicating the successful development of a high affinity sensor method (Fig. 6). Also, the results of competitive assay confirmed that the binding of the AuNP conjugated target on the nanoMIP surface occurred via the interaction between vancomycin and the surface receptor.

**Figure 7:**
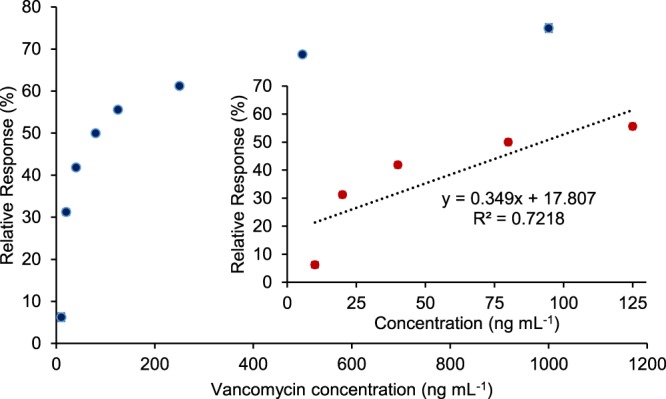
The proportional relationship between the free vancomycin concentrations and the sensor signal, and linear calibration plot of the vancomycin nanoMIP based sensor for competitive assay.

To calculate the LOD of competitive assay, the sensor signals in Fig. 6B were initially converted to relative signal (%) to obtain the proportional relationship between the free vancomycin concentrations and the sensor response (Fig. 7). The relative signal for each free vancomycin concentration was calculated based on Equation 1:

1 $${\rm{Relative}}\,{\rm{Response}}\,[ \% ]=\,(\frac{{\rm{Conjugated}}\,{\rm{vancomycin}}\,{\rm{signal}}-{\rm{Total}}\,{\rm{signal}}\,{\rm{of}}\,{\rm{conjugated}}\,{\rm{and}}\,{\rm{free}}\,{\rm{vancomycin}}}{{\rm{Conjugated}}\,{\rm{vancomycin}}\,{\rm{signal}}})\,\cdot \mathrm{100} \% $$

The conjugated vancomycin without the addition of free vancomycin produced 160 RU response, which is 10 RU higher than that of the sample included the lowest free vancomycin concentration (10 ng mL^−1^) in addition to the conjugated vancomycin (150 RU). The Scatter graph in the concentration range of 10–1000 ng mL^−1^ was plotted (Fig. 7) and the LOD of the competitive assay was determined based on the linear portion of this plot (Fig. 7, inset) by calculating three times standard deviation of the blank response (3 × 8 = 24 RU). The LOD was determined to be 17.7 ng mL^−1^ with a linear range from 10 ng mL to 125 ng mL^−1^. The competitive assay can be used as an alternative method for antibiotic detection in milk samples. However, it is 1.77 time less sensitive than the threshold MRL concentration of antibiotics for milk (10 µg kg^−1^). The sensitivity of direct assay is 4.3 times higher than that of competitive assay. It is also worth mentioning that the reproducibility of the direct assay (R^2^ = 0.9697) is far better than the competitive assays (R^2^ = 0.7218) that is an important factor for the accuracy and reliability of the detection method.

To verify the accuracy of the sensor methods, the recovery studies were performed with a known quantity of vancomycin, which was previously determined for sensor methods, in the presence of milk. The samples were measured with sensor using direct and competitive assays before and after the addition of 5, 15 and 35 ng mL^−1^ of vancomycin. The recoveries values for direct assay were determined to be 110%, 98% and 96% for 10, 20 and 40 ng mL^−1^ concentrations of vancomycin respectively with acceptable relative standard deviations (RSDs) below 3%. The slightly lower recoveries rates, ranging between 85% and 110%, were obtained with competitive assay with relatively higher RSDs (Table 3). All the recoveries rates were comprised between 80% and 110%^32^ (reference value suggested by the Association of Analytical Communities—AOAC), suggesting that both methods can reliably be used for milk sample analysis. However, the nanoMIP-SPR sensor using direct assay demonstrated higher accuracy and can be applied very well for the analysis of this kind of sample without significant matrix effect.

**Table 3:** The recoveries rates of direct and competitive assays for milk samples spiked with vancomycin (n = 3).

Contained vancomycin (ng mL^−1^)	Added vancomycin (ng mL^−1^)	Recovered vancomycin (ng mL^−1^)		Recovery (%) ± RSD	
		DA	CA	DA	CA
5	5	11.0	9.5	110 ± 2	95 ± 6
5	15	19.6	17	98 ± 2	85 ± 7
5	35	38.5	35	96 ± 2	88 ± 7

DA: Direct assay; CA: Competitive assay.

### Comparison of NanoMIP and NIP

**Figure 8:**
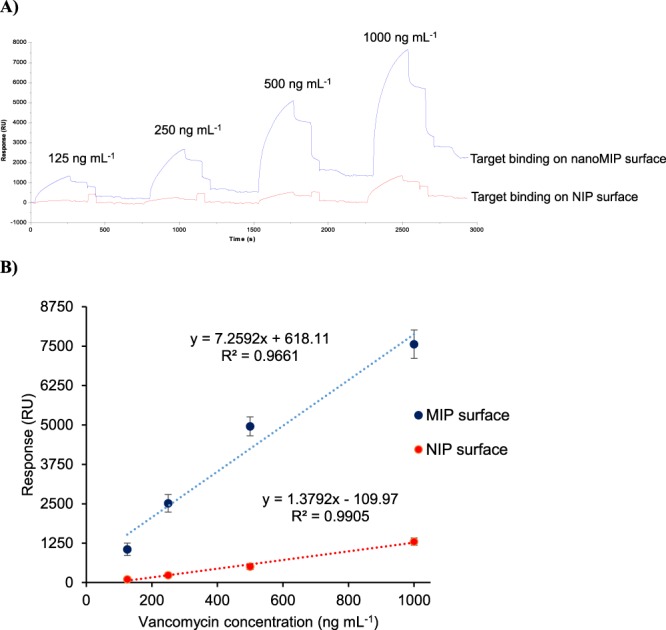
Real time SPR sensorgram of vancomycin binding on nanoMIP and NIP immobilised surfaces (**A**). Comparison of overall nanoMIP and NIP assays (n = 3) (**B**).

This study has comparatively investigated the binding of vancomycin on non-imprinted polymer (NIP) and nanoMIP surfaces in the concentration range of 125–1000 ng mL^−1^ to further determine the specificity of the nanostructured polymeric receptors towards the target molecule. Each sample was injected onto two separate sensor surfaces for 4 min. Despite the surface could not be completely regenerated for subsequent analysis of different vancomycin concentrations in these assays, a significant difference was recorded for analyte binding on NIP and nanoMIP surfaces. The real time sensorgram of this test is shown in Fig. 8A. The data of three parallel experiments were subjected to the linear regression analysis that resulted in R^2^ values of 0.96 and 0.99 for nanoMIP and NIP, respectively (Fig. 8B). The imprinting factor was calculated based on the average sensor signals in the entire concentration range and it was determined as 7.4. An electro-synthesised MIP prepared for the detection of analgesic drug aminopyrine provided an imprinting factor of 6.67 and a detection limit of 0.13 mg mL^−1^ ^15^. The MIP monolith, prepared by *in situ* thermal-initiated polymerisation for the determination of pefloxain (an antibacterial drug) from milk samples, was coupled with an HPLC system that revealed an imprinting factor of 3.1 and a limit of quantification of 4.7 ng mL^−1^. However, HPLC is a labour intensive technique and the sample pre-treatment was required^33^.

## Materials and Methods

### Materials and Reagents

3-aminopropyltrimethyloxysilane (APTMS), glutaraldehyde, vancomycin hydrochloride, artemisinin, teicoplanin, N,N-diethyldithiocarbamate acid benzyl ester, itaconic acid, pentaerylthirtol tetrakis(3-mercaptopropionte) 95% (chain transfer agent: CTA), trimethylolpropane trimethacrylate (TRIM), ethylene glycol dimethacrylate (EDGMA), acetonitrile, 60 mL SPE tubes and 20 μm pore frits, N-(2-Hydroxyethyl)piperazine-N′-(2-ethanesulfonic acid) (HEPES) buffer, 11-mercaptoundecanoic acid (MUDA) 95%, N-hydroxysuccinimide (NHS), 1-ethyl-3-(3-dimethylaminopropyl) carbodiimide (EDC), ethanolamine hydrochloride, gold nanoparticles (Au-NP), toluene, chloroform, 2-morpholinoethanesulfonic acid monohydrate (MES), N-(3-aminopropyl)methylacrylamide hydrochloride and phosphate buffered saline (PBS) were all obtained from Sigma Aldrich. Sodium hydroxide (NaOH) was purchased from VWR. Hexaferrocyanide (~ 99%, K_3_[Fe(CN)_6_]), potassium chloride (KCl) absolute ethanol were purchased from Roth. Micro glass beads were purchased from Blagden chemicals. Double-distilled ultrapure water was used for analyses.

### Instrumentation and Apparatus

The size and stability of nanoMIPs were measured using dynamic light scattering (DLS) analysis with a Malvern Zetasizer NanoS instrument (Malvern Instruments). A fully automated SPR-based Biacore 3000 sensor (Uppsala, Sweden) was used for target detection assays and cross-reactivity studies. Bare gold sensor chips were obtained from Biacore GE Healthcare (Uppsala, Sweden). Surface characterisation studies for bare gold chip, MUDA coated chip and MIP immobilised chips were performed using PalmSens4 compact electrochemical interface with a three-electrode electrochemical system that was configured by connecting a gold working electrode, an Ag/AgCI reference electrode and a platinum wire as counter electrode in a cell with a volume of 5 mL (Belltec, Netherlands). Operating temperature of all instruments was 25 °C for the experiments. A sonicator and vortex from VWR (Dresden, Germany), were employed to dissolve the compounds.

### Rational Design of NanoMIPs Using Molecular Modelling

The workstation used for rational design of the synthetic receptors was an RM tower running Linux 6.2 operating system. The workstation was configured with 3.20 GHz Intel® Core TM i5-3470 processor, 320 GB hard drive and 8 GB RAM. This system was utilised for the software package SYBYL Tripos (St. Louis, Michigan, USA). The 2D structure of vancomycin was attained from the drug database. It was converted to 3D structure for minimisation. The molecule was then charged by the Gasteiger–Huckel method and the molecular mechanics was applied to minimise the structure using the Powell method. The minimisation was run until the convergence gradient reached 0.001 kcal mol^−1^. The procedure was fully described elsewhere^13^. A virtual library of 25 monomers characteristically used in molecular imprinting was screened using the LEAPFROG™ algorithm that exists within the modelling software^13,22,23^. The algorithm selected monomers according to the strongest binding interactions with the vancomycin target and then minimised in organic solvents. The binding energy results were scaled from the highest binding scores to the lowest. The binding interactions between each functional monomer and the target template can be visualised by employing the software.

### Preparation of Template Immobilised Solid Supports

Micro glass beads (60 g per production, 45 μm < diameter < 90 μm) were vibrated with abrasive ceramic beads to eliminate any surface coating. The exposed surfaces of micro beads were then activated in 1 M NaOH during 10 min before thoroughly washing with double-distilled water at 60 °C. Another washing step was carried out with acetone at room temperature and the beads were then kept in an oven to dry (80 °C, 2 h). The dry glass beads were incubated overnight in a 2% v/v solution of 3-aminopropyltrimethyloxysilane (APTMS) in toluene at 4 °C to introduce amino groups to their surfaces. Subsequently the solution was removed, the glass beads were washed with double distilled water and dried for 1 h using the vacuum^25^.

To enable the binding of vancomycin template to the glass beads, aldehyde groups are required. Therefore, glutaraldehyde was coated on the silica surface of the glass beads. The aldehyde group of gluteraldehyde is able to make a covalent bond with an amine group of vancomycin, namely a peptide bond^21^. The activation step was performed by incubating 60 g of glass beads together with 50 mL of a phosphate buffered saline (PBS)- glutaraldehyde mixture during 2 h at room temperature. Hereof, 7% PBS has to be replaced by glutaraldehyde. Later on, the beads were washed with double-distilled water to remove the excess of gluteraldehyde. Subsequently, a 0.5 mg mL^−1^ vancomycin prepared in PBS (50 mL, pH 7.4) was added to the glass beads. The glass beads were incubated overnight (4 °C) and unbound vancomycin was washed using deionised water followed by 15 min incubation with ethanolamine (50 mL, 0.01 M) to cap any unreacted groups on glass bead to prevent from non-specific binding during the polymerisation. The template immobilised micro glass beads were rinsed with double distilled water and dried under vacuum, then stored at 4 °C.

### NanoMIP Synthesis

The polymerisation mixture was prepared by mixing 2.18 g of itaconic acid as functional monomer, 3.24 g ethylene glycol dimethacrylate (EDGMA) and 3.24 g of trimethylolpropane trimethacrylate (TRIM) as cross-linkers, 0.18 g pentaerylthirtol tetrakis(3-mercaptopropionte) (CTA) as chain transfer agent and 0.753 g N,N-diethyldithiocarbamate acid benzyl ester as iniferter. The iniferter has three functions in the polymerisation process: initiator, transfer agent and terminator. All compounds were dissolved in acetonitrile (10.52 g). The polymerisation mixture placed in a glass vial was purged with nitrogen for 20 min. 60 g of vancomycin derivatised glass beads was placed in 200 mL flat glass beaker and degassed under vacuum for 15 min. The polymerisation solution was poured onto the glass beads that allow the functional monomer to bind to the vancomycin template via electrostatic interactions followed by polymerisation in the presence of the initiator. Subsequently the mixture was exposed to UV light that triggered the polymerisation reaction. The exposure to UV light is a critical step in the production process and must be exactly 2 min. To add carboxyl group on the MIP, a 30 s polymerization was further performed using the secondary monomer [N-(3-aminopropyl) methacrylamide hydrochloride] solution (5 mg monomer was dissolved in 7 mL of acetonitrile and then poured over the solid supports). Addition of carboxyl group to the MIP surface is essential for sensor construction, because nanoMIPs need to be covalently immobilised to the sensor chip surface^9^. After polymerisation the glass beads were transferred into SPE cartridge and washed six times using cold acetonitrile (0 °C) in an ice bath. Cold wash was carried out in order to remove low affinity polymeric structures and fragments. As the next step, the SPE cartridge was incubated in hot acetonitrile (60 °C) for 6 min and the high affinity nanoMIPs were then eluted from the solid phase. The last step was repeated five times to obtain a total volume of 150 mL high fraction of nanoMIPs. Non-imprinted polymer was also synthesised under the same conditions in the absence of target molecules to investigate the specificity of nanoMIPs for vancomycin target in terms of size, shape and chemical functionality of the cavities.

### DLS Characterization of NanoMIPs

The size, stability and quality of nanoMIPs were determined by using the DLS method. DLS is also called as photon correlation spectroscopy and enables to determine the size and the distribution profile of particles and polymers in a solution. The measurements were realised using disposable polystyrene cuvettes of 3 cm^3^, along with a Zetasizer Nano (Nano-S) at 25 °C. Before the analysis, the nanoMIP samples were prepared in 1 mL volume, sonicated for one minute and filtered through a 0.22 μm glass fibre filter, respectively.

### NanoMIP-SPR Sensor for Bio-detection Assays

#### Sensor chip cleaning and SAM formation

SPR sensor chips were cleaned using piranha solution. The chips were initially washed with ethanol and double distilled water, respectively. The piranha solution was prepared by pouring one part of hydrogen peroxide (H_2_O_2_) to three parts of sulphuric acid (H_2_SO_4_) in a glass vessel. The chips were immersed in this solution for 15 min. The solution was then removed after diluting it in 4 mL of double distilled water and the chips were washed with water until a pH of 6.0 was observed. Later on, the sensor chips were further rinsed with ethanol to remove all possible contaminants from the surface. After the cleaning process the SPR chips were directly immersed in 2 mM MUDA solution prepared in absolute ethanol to form a self-assembled monolayer on the surface during overnight incubation.

#### Vancomycin detection assays using nanoMIP SPR sensor

MUDA coated sensor chips were docked to Biacore 3000 system and primed with HEPES buffer (10 mM, pH 7.4). All sensor assays were carried out at room temperature using HEPES solution as the running buffer at a flow rate of 10 µL min^−1^. The MUDA coated surface was activated by injecting the mixture of EDC (0.4 M) and NHS (0.1 M) in 1:1 volume ratio for 4 min. A 500 μg mL^−1^ of nanoMIPs in MES (pH 6.5), previously concentrated by evaporation, was then covalently attached to the surface during two subsequent injections (5 min per each injection). The nanoMIP-free surfaces were blocked by subsequent injection of ethanolamine (0.1 M) for 4 min. Vancomycin samples were then prepared in milk and each sample was injected to the sensing surface for 4 min from the lowest concentration to the highest. The milk was defatted as described by Paniel *et al*.^34^ and the skimmed milk was used to prepare the vancomycin samples in the concentration range of 5–1000 ng mL^−1^. Kinetic data analysis was performed using Biacore 3000 analyzer to determine the affinity between nanoMIPs and vancomycin target, and it was expressed as dissociation constant (K_d_).

#### Conjugation of pharmaceuticals with gold nanoparticles as signal amplification agents

Drugs are small molecules and their sensitive detection by sensors commonly requires the use of nanomaterials. Therefore, vancomycin was conjugated with gold nanoparticles to detect trace amounts of the drug in milk samples. The conjugation procedure was previously described in detail by Altintas *et al*.^9^. For cross-reactivity studies, non-specific drug molecules (artemisinin and teicoplanin) were also conjugated with AuNPs under the same conditions.

### Sensor Surface Characterisation Using Voltammetry Techniques

The bare, MUDA coated and nanoMIP immobilised sensor surfaces were characterised using voltammetry techniques. For this PalmSens4 compact electrochemical interface with a three-electrode electrochemical system was used by connecting SPR chip as a gold working electrode, an Ag/AgCI reference electrode and a platinum wire as auxiliary electrode in a cell with a volume of 5 mL. A 0.1 M ferrocyanide in 1 M KCl was used for cyclic voltammetry (CV) and square wave voltammetry (SWV) measurements. The potentials were cycled from −0.2 to + 0.8 V at a scan rate of 50 mV/s for CV. The applied potentials for SWV were from −0.2 to + 0.8 V at a step height of 3 mV, an amplitude of 0.05 V, and a frequency of 10 Hz. All measurements were conducted at room temperature.

## Conclusions

A new nanoMIP-SPR sensor for the detection of vancomycin in milk has been reported. NanoMIPs targeting vancomycin were rationally designed by employing computational simulations that enormously reduced the experimental screening time in the laboratory. The synthetic receptors were capable of detecting vancomycin in the range of 10–1000 ng mL^−1^ with high sensitivity and selectivity using an optical sensor. Taking into account that the MRL for antibiotics in milk ranges from 10 to 200 µg kg^−1^, the sensor demonstrates higher sensitivity than the threshold MRL level. The comparative investigations on nanoMIPs and NIPs demonstrated the great selectivity of nanoMIPs towards their target molecule in terms of shape, size and chemical functionality and this was confirmed by an imprinting factor of 7.4. Cross-reactivity studies with other pharmaceuticals showed high specificity of nanoMIPs towards vancomycin. The affinity between the target drug and the synthetic receptors was found to be 1.8 × 10^−9^ M that is mostly higher than those of natural and other synthetic receptors for vancomycin. The use of nanoMIP sensors in the field of food sample analysis for the detection of important contaminants offers cheap, easy-to-apply, reliable and fast bio-detection methods. The nanoMIP SPR may have immense impact both in food and health sectors.

## Electronic supplementary material

Supplemantary Information
