# Supplementary material for: Surface plasmon resonance based sensor for the detection of glycopeptide antibiotics in milk using rationally designed nanoMIPs
Source: Sci Rep. 2018 Jul 25;8:11222. doi: 10.1038/s41598-018-29585-2 (PMC6060165; doi:10.1038/s41598-018-29585-2)
Supplement: Supplementary file 1 — Supplemantary Information [file 41598_2018_29585_MOESM1_ESM.pdf]

Supplementary Information

Surface plasmon resonance based sensor for the detection of glycopeptide antibiotics in milk using rationally designed nanoMIPs

Zeynep Altintas*

Institute of Chemistry, Technical University of Berlin, 10623, Germany

*zeynep.altintas@tu-berlin.de

*Corresponding Author:

Zeynep Altintas PhD

Institute of Chemistry, Faculty of Maths and Natural Sciences, Straße des 17. Juni 124, Berlin 10623, Germany

E-mail: zeynep.altintas@tu-berlin.de

Tel: +49 30 314 23727

Fax: +49 30 314 79552

Visualisation of vancomycin-monomer interactions using computational modelling

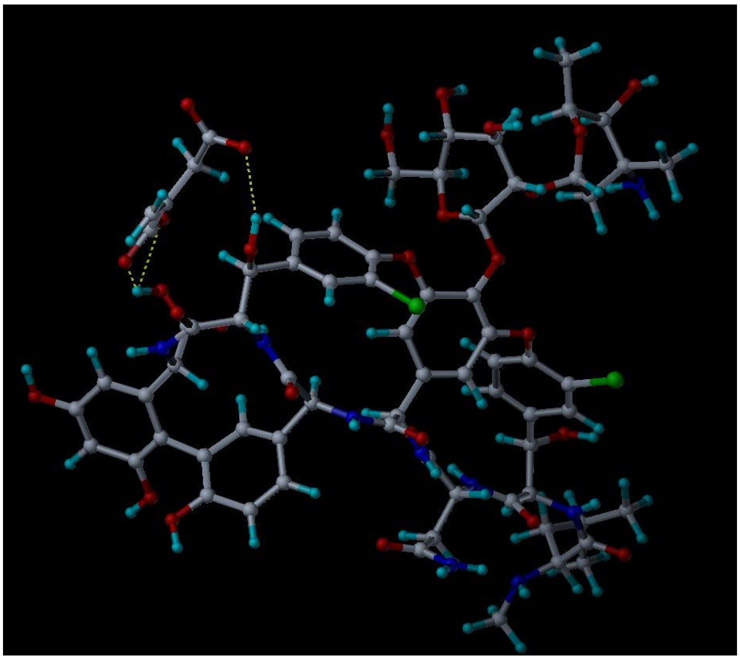

Figure S1: Visualisation of binding interaction between vancomycin and itaconic acid.

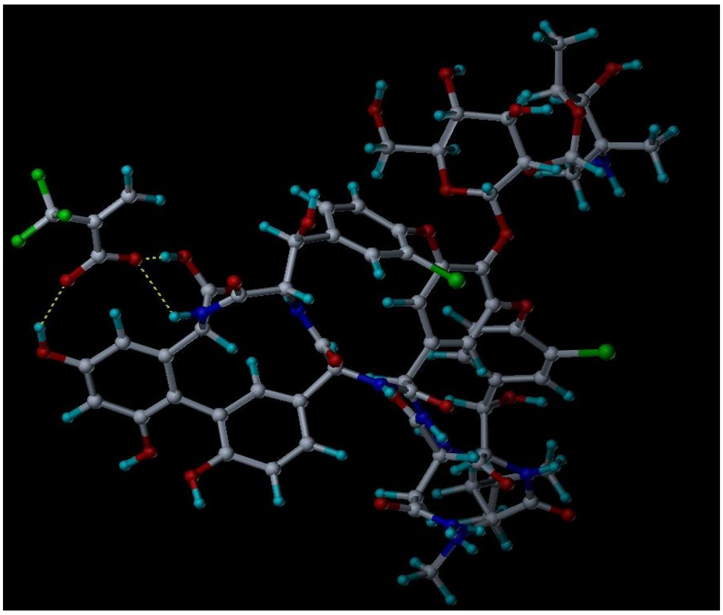

Figure S2: Visualisation of binding interaction between vancomycin and trifluoromethacrylic acid.

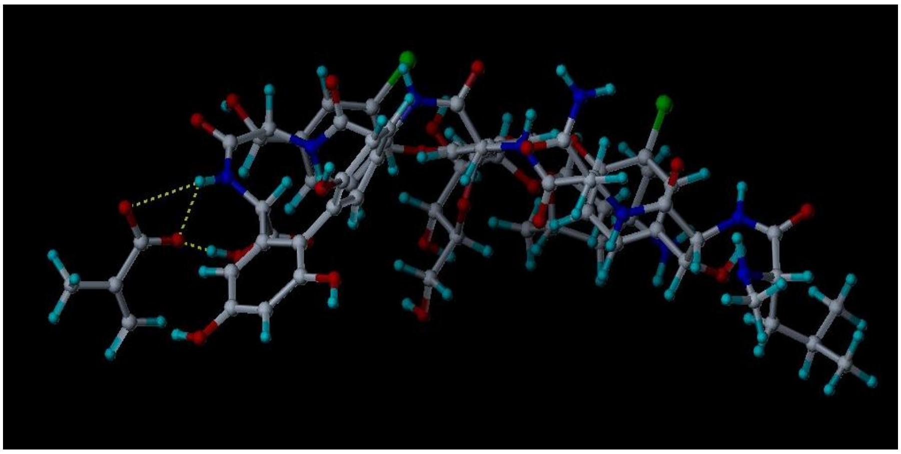

Figure S3: Visualisation of binding interaction between vancomycin and methacrylic acid.

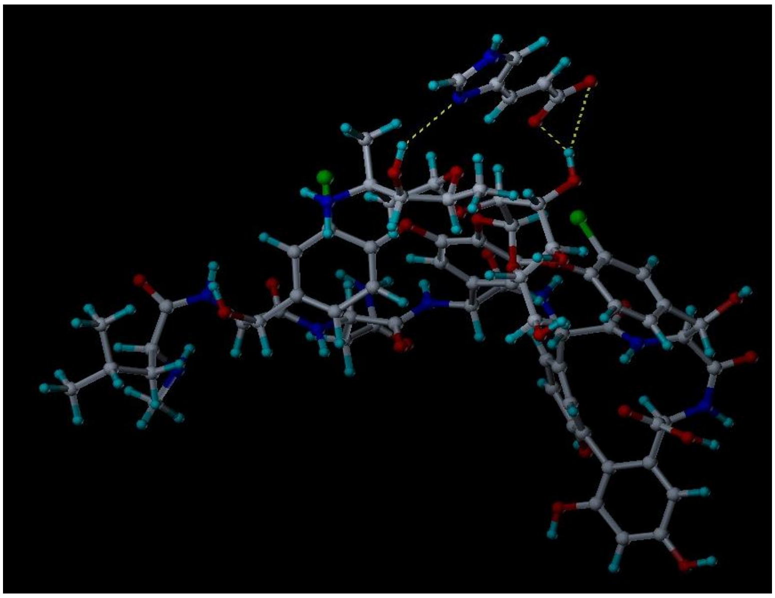

Figure S4: Visualisation of binding interaction between vancomycin and urocanic acid.

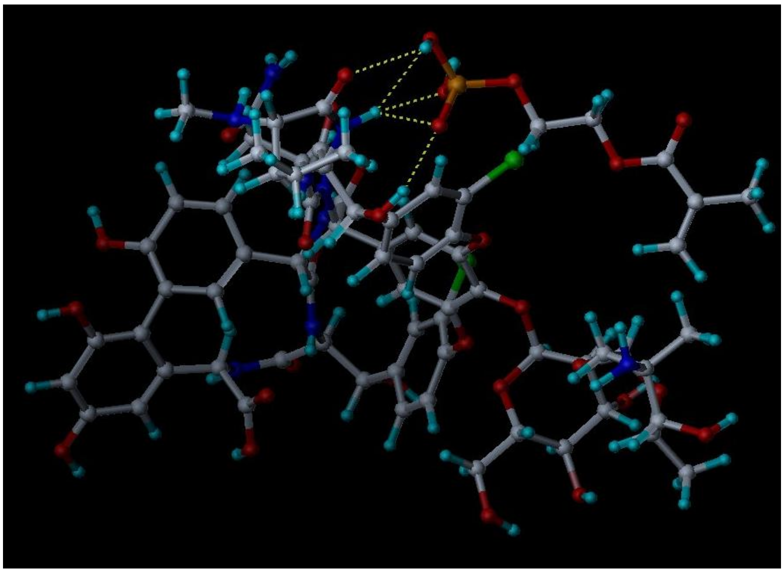

Figure S5: Visualisation of binding interaction between vancomycin and ethylene glycol methachrylate phosphate.

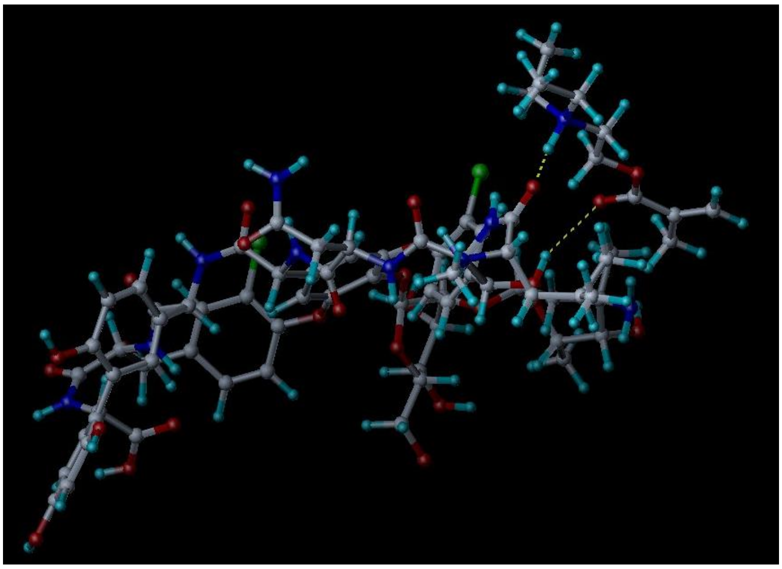

Figure S6: Visualisation of binding interaction between vancomycin and diethylaminoethyl methacrylate.
